# Characteristics of Top-Searched Individuals in Japan’s Yen for Docs Conflicts of Interest Database During the COVID-19 Pandemic

**DOI:** 10.7759/cureus.47264

**Published:** 2023-10-18

**Authors:** Yudai Kaneda, Akihiko Ozaki, Takanao Hashimoto, Yosuke Suzuki, Hiroaki Saito, Tetsuya Tanimoto, Erika Yamashita, Mihajlo Jakovljevic

**Affiliations:** 1 Medicine, Hokkaido University, Sapporo, JPN; 2 Breast and Thyroid Surgery, Jyoban Hospital of Tokiwa Foundation, Fukushima, JPN; 3 Public Health, Medical Governance Research Institute, Tokyo, JPN; 4 Pharmacy, Kenkodo Pharmacy, Osaki, JPN; 5 Gastroenterology, Soma Central Hospital, Soma, JPN; 6 Internal Medicine, Navitas Clinic, Tokyo, JPN; 7 Heart Care, Medical Governance Research Institute, Tokyo, JPN; 8 Comparative Economic Studies, Hosei University, Tokyo, JPN; 9 Global Health Economics and Policy, University of Kragujevac, Kragujevac, SRB; 10 Advanced Manufacturing Technologies, Peter the Great St. Petersburg Polytechnic University, St. Petersburg, RUS

**Keywords:** japan, yen for docs database, number of searches, conflicts of interest, covid-19

## Abstract

Purpose

Transparency in healthcare has led to increased public disclosure of doctors' conflicts of interest, with the "Yen for Docs Database" in Japan emerging as a pivotal source. Nevertheless, there remains ambiguity regarding the backgrounds and influence of highly-searched persons, especially during the COVID-19 pandemic. The primary objective of this study was to examine if the database was utilized for its intended purpose in 2021, a year marked by the introduction of vaccines and treatments, the addition of new COVID-19-related data, and the frequent appearances of expert statements in various media outlets.

Methods

We conducted a descriptive analysis on the 10 most frequently searched individuals in the "Yen for Docs Database" between August 27 and September 23, 2021, and determined the amount of money they received from pharmaceutical companies and other organizations over the four-year period between 2016 and 2019. To characterize frequently searched individuals' academic profiles and appearances in the mass media, we identified their h-index and affiliation, their activity on Twitter, and the number of TV appearances.

Results

There were 72,904 searches during the study period, with the top person accounting for 4,905 of those searches. All top 10 were male, mostly affiliated with universities and specialists in infectious diseases or related fields. Their median number of COVID-19 articles was five, and the median h-index was 34. Four of these top 10 had Twitter accounts, with followers ranging from 12,000 to 195,000. The median amount received from pharmaceutical entities over four years was $154,930, ranging from $809 to $705,502.

Conclusions

In the Yen for Docs Database, a significant portion of searches during the COVID-19 pandemic was concentrated on a selected group of healthcare professionals with considerable payments over the years, and they exhibited prominent academic and media profiles. These observations highlight the need for more transparent conflicts of interest disclosure among physicians with public visibility.

## Introduction

The primary interest of healthcare providers (HCPs) and healthcare organizations (HCOs) is the health and well-being of their patients, with patient-centered care being a core concept of modern medicine [1,2]. However, financial relationships with pharmaceutical companies are significant sources of financial conflicts of interest (FCOI) for HCPs and HCOs [3-6], making the management of such relationships a crucial aspect of health policy. Given that the promotion of transparency instead of outright banning interactions is a practical approach, many high-income countries conduct transparency initiatives grounded in self-regulation or legal frameworks [7-12]. Further, some countries have implemented open-access databases integrating such information, including the Open Payments Database in the United States, mandated by the Sunshine Act [13], and Disclosure UK [14]. Alongside these, non-profit initiatives like the Dollars for Docs by the ProPublica in the United States have emerged [15].

Despite these efforts, public access to information regarding financial relationships is often constrained by data quality and accessibility in many countries [16,17]. For instance, in Japan, the third-largest pharmaceutical market globally, pharmaceutical companies only disclose certain payments, limiting transparency and accessibility to the general population [18,19]. This lack of standardization and openness makes it difficult for patients and citizens to access accurate information, signifying the necessity for unified systems [20]. The Medical Governance Research Institute has collected and organized the payment data disclosed by the major pharmaceutical companies and has launched the Yen for Docs Database, an open-access payment data similar to the Dollars for Docs, which has been accessed by approximately 31,000 people as of October 2021 [21].

While these public databases have improved transparency regarding financial relationships between pharmaceutical companies and healthcare entities [11], questions often arise concerning their practical impact [22]. For example, public awareness remains low, as demonstrated in the United States, where less than 10% of respondents knew whether their physicians received industry payments [22].

Against the backdrop of the novel COVID-19 pandemic, there arises a distinctive opportunity to ascertain whether open-access payment databases are fulfilling their foundational objective: enhancing public access to pharmaceutical payment data [23]. During the COVID-19 pandemic, diverse observations were posited by experts across mediums, including television, newspapers, and Twitter in Japan [24-26]. However, there exists an opacity regarding the financial ties of influencers and specialists featured on mainstream media and social networking platforms, as these platforms are not interested in disclosing such information. In such a milieu, it is anticipated that the general public will rely heavily on open-access databases, such as the Yen for Docs Database, during the COVID-19 pandemic [24].

The overarching aim of this study was to investigate whether the Database is being used for its intended purpose in 2021, a year marked by the emergence of vaccines and treatments. The overall access to the Yen for Docs Database and the characteristics of those who were searched the most frequently were examined in detail.

## Materials and methods

Study settings

The search history of the Yen for Docs Database from August 27, 2021, to September 23, 2021, was retrieved, and a ranking of the top 10 most frequently searched individuals was obtained. We investigated and extracted information regarding their gender, affiliation, medical license status, specialty, number of COVID-19-related articles, h-index, use of social networking sites (SNS), number of television appearances in the first half of 2021, and the amount of individual income received from pharmaceutical companies between 2016 to 2019. Recognizing that SNS platforms, particularly LINE, which are popular in Japan and, as our previous research has indicated, influence shifts in intentions to receive the COVID-19 vaccine, are often utilized more for personal communication than for disseminating information [27]. Therefore, in this study, we specifically examined their usage of Twitter, a platform better suited for reaching a wider audience.

Data collection

The data pertaining to the number of TV appearances were collected by Japan Monitor, encompassing the period from January 1 to June 20, 2021, with only the top 10 appearances made available. The number of articles related to COVID-19 was obtained by conducting a search using the author's name in conjunction with keywords such as "COVID-19," "SARS-CoV-2," or "coronavirus" on PubMed, up until October 2021. Affiliations and h-indexes were determined by searching the authors' names in Scopus and extracting relevant information, also as of October 2021. Additional information was gathered by performing a direct Google search using their names. This data was then subjected to descriptive analysis.

Regarding Twitter usage, the authors' names were entered directly into the Twitter application to ascertain the existence of open public accounts. If Twitter accounts were identified, the number of followers was recorded as of October 28, 2021, along with the count of tweets made by the account holders themselves during the week spanning October 22-28, 2021. It should be noted that the count excluded the number of retweets and replies.

It should be noted that all personal names in the collected data were anonymized. Individuals were labeled in alphabetical order based on the frequency of search queries, designated as Individual A, B, C, and so forth.

Analysis

We conducted a descriptive analysis of the collected data. Furthermore, for the top 10 healthcare professionals with the highest number of searches, we computed the mean and median values concerning their number of articles related to COVID-19, h-index, and pharmaceutical payments (encompassing speeches, publishing, and consulting) spanning the years 2016 to 2019. These calculations were performed using Microsoft Excel 2016 (Microsoft Corporation, Washington, United States).

Ethical statement

The Ethics Committee of the Medical Governance Research Institute approved this study (approval number: MG2018-04-20200605; approval date: June 5, 2020) and waived to gain informed consent from study participants because all data in this study were publicly available.

## Results

Table 1 shows the characteristics of the top 10 individuals searched most frequently in the Yen for Docs Database during the study period.

**Table 1:** Characteristics of the top 10 persons searched most frequently

No.	Name (Anonymized)	Affiliation	Search Count	Gender	Medical License	Specialty	Number of Corona-Related Papers	H-INDEX
1	Individual A	Osaka University Professor	4905	Male	Yes	Infectious Diseases	34	17
2	Individual B	Mayor of Yokohama City	4349	Male	No	Data Science	5	39
3	Individual C	International University of Health and Welfare Professor	2446	Male	Yes	Infectious Disease & Infection Control	1	35
4	Individual D	Showa University Visiting Professor	2312	Male	Yes	Infectious Diseases & Respiratory	0	33
5	Individual E	Aichi Medical University Professor	1764	Male	Yes	Infectious Diseases	5	23
6	Individual F	Nagasaki University Professor	1496	Male	Yes	Pediatrics	4	35
7	Individual G	Toho University Professor	852	Male	Yes	Medical Microbiology	11	43
8	Individual H	COV-Navi	838	Male	Yes	Epidemiology	7	7
9	Individual I	Nagasaki International University Professor	507	Male	Yes	Neurology	0	59
10	Individual J	Interpark Kuramochi Respiratory Internal Clinic Director	501	Male	Yes	Respiratory Internal Medicine	5	5

Of the total 72,904 searches made during this period, the most frequented individual had 4,905 searches, followed by 4,349, 2,446, and 2,312 searches, respectively. Of the total searches, the top 10 accounted for 27.4%. All the people in the ranking were male, and all but Individual B had a medical license. Seven out of 10 were professors affiliated with universities, and four of them specialized in infectious diseases, with the others in statistics (data science), pediatrics, microbiology, neurology, and respiratory medicine. The median number of COVID-19-related articles per person for these individuals was five (IQR: 1-7), and the median h-index was 34 (IQR: 17-39).

The total amount of money they received during the four years from 2016 to 2019 was highest for Individual E at $705,502, followed by Individual G and D at $314,826 and $279,657, respectively. The lowest was Individual H's $809, followed by Individual J's $1,876. The mean and median of the total amount of money they received during the four years were $193,940 (SD: $198,211) and $154,930 (IQR: $35,149-279,657), respectively.

**Figure 1:**
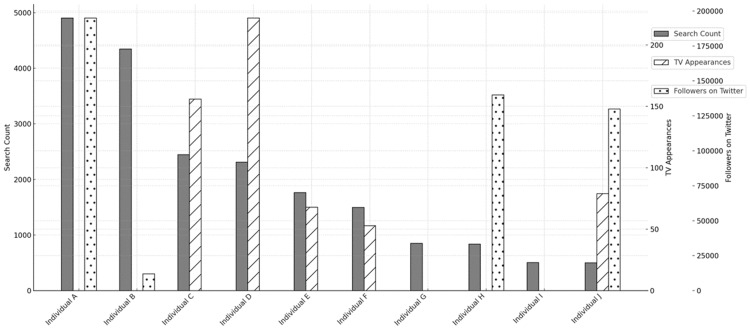
Comparison of search counts, TV appearances, and followers on Twitter

Figure 1 illustrates the comparison of their search counts, TV appearances, and Twitter followers.

We found four Twitter accounts of the top 10 healthcare professionals with the most frequent searches: Individuals A, B, H, and J. Each of them had about 195,000 followers, 12,000 followers, 140,000 followers, and 130,000 followers as of October 28, 2021, respectively. The number of tweets that these individuals had made on their own in the past week was 3, 0, 56, and 20, respectively. Additionally, in the first half of 2021, Individual D made the most appearances on TV (222 times), followed by Individual C (156 times) and Individual (79 times).

## Discussion

Through the analysis, it was determined that during the research period under the COVID-19 pandemic, it was ascertained that the experts ranking high in terms of access counts were primarily those directly involved in fields relating to infectious diseases and the pandemic. Additionally, these specialists were found to be prominent figures on media platforms like Twitter and television, thereby underscoring their significant social influence. Against this background, the Yen for Docs Database was elucidated to be a broadly utilized search database by the general public, particularly in scenarios such as the COVID-19 pandemic, where the concerted efforts of diverse field experts are requisite.

While these professionals play a critical role, their financial relationships with HCOs must be scrutinized. The data revealed that several top-ranked individuals received substantial payments from pharmaceutical companies, particularly from the time period year 2016 to 2018. Although these financial relationships are not inherently illegal, they can lead to socially and politically problematic situations, including the potential for biased statements [28].

The study also found that healthcare professionals frequently appearing on TV and active on social media were often searched in the database. In particular, four of the top 10 healthcare professionals had significant Twitter followings, ranging from 12,000 to 195,000 followers. Although these platforms substantially impact public opinion, they also come with risks, such as spreading misinformation and the lack of regulation for disclosing FCOI [29].

The connection between healthcare professionals and pharmaceutical payments is not simple [30]. For instance, professionals like Dr. Shigeru Omi, the former president of the Subcommittee on Novel Coronavirus Disease Control, had a significant impact in determining Japan's COVID-19 countermeasures [31]. However, members of this committee may have potential financial and non-financial conflicts of interest with pharmaceutical companies such as AstraZeneca, Pfizer, and Moderna, which played a pivotal role in supporting Japan's vaccine strategy, necessitating a need to scrutinize underlying biases [32,33]. Remarkably, as Individual B is the mayor of one of Japan's largest cities, his financial relationships with the industry are particularly concerning. Given the indications that messages from policymakers can influence individuals' health behaviors and the responses of medical institutions, it is imperative that such communications maintain a high level of transparency [34,35].

The results also emphasize the need for more stringent regulations regarding the disclosure of FCOI in media and social networking services [36]. Considering the potential subconscious influence of FCOI on medical professionals' decisions, there is a call for transparency in financial relationships, significantly when such influence can shape public policies and opinions [37,38].

This study has some limitations, including the focus on the top 10 individuals, which may not provide a comprehensive view. Additionally, the manual counting of tweets and articles may have introduced some errors. However, these findings not only provide a valuable starting point for further research and potential regulatory considerations but also offer a significant perspective on Japan's adherence to its traditional principle of infallibility in COVID-19 measures, possibly illuminating the rationale behind such unwavering strategies [39-41].

## Conclusions

This study revealed that during the COVID-19 pandemic, particularly influential medical professionals were frequently searched in the Yen for Docs Database. In a world where medical professionals are increasingly engaging with the public through various channels, a clear understanding of their affiliations, biases, and the potential influence of financial interests becomes crucial. Despite not being inherently problematic, these financial ties necessitate greater transparency and stricter regulations to prevent potential biases.
